# Daily Sedation Interruption vs Continuous Sedation in Pediatric Patients Receiving Mechanical Ventilation: A Systematic Review and Meta-analysis

**DOI:** 10.1001/jamanetworkopen.2024.26225

**Published:** 2024-08-07

**Authors:** Theresa Shu Wen Toh, Pravin R. R., Karen Hwee Ying Ho, Rehena Sultana, Rachel Couban, Karen Choong, Jan Hau Lee

**Affiliations:** 1 Department of Pediatric Subspecialties, Children’s Intensive Care Unit, KK Women’s and Children’s Hospital, Singapore; 2 Department of Pediatric Medicine, KK Women’s and Children’s Hospital, Singapore; 3 Center for Quantitative Medicine, Duke-NUS Medical School, The Academia, Singapore; 4 Department of Anesthesia, McMaster University, Hamilton, Ontario, Canada; 5 Department of Pediatrics, McMaster University, Hamilton, Ontario, Canada; 6 Department of Critical Care, McMaster University, Hamilton, Ontario, Canada; 7 Department of Health Research Methods, Evidence and Impact, McMaster University, Hamilton, Ontario, Canada; 8 Paediatrics Academic Clinical Program, Duke-NUS Medical School, Singapore

## Abstract

**Importance:**

The effectiveness of daily sedation interruption (DSI, defined as temporary interruption of sedation) has yet to be demonstrated in critically ill pediatric patients.

**Objective:**

To compare the clinical outcomes of DSI vs continuous intravenous (IV) sedation in patients receiving invasive mechanical ventilation (MV) support in the pediatric intensive care unit (PICU).

**Data Sources:**

A systematic search for studies was conducted using predefined keywords and Medical Subject Headings in 5 major databases (PubMed, Embase, Web of Science, CINAHL [Cumulated Index to Nursing and Allied Health Literature], and Cochrane Central Register of Controlled Trials) from database inception to October 31, 2023.

**Study Selection:**

Retrospective and prospective observational studies, randomized clinical trials (RCTs), and systematic reviews were assessed for inclusion. Studies were eligible if they compared DSI to continuous IV sedation in patients aged 18 years or younger requiring MV in the PICU.

**Data Extraction and Synthesis:**

Study characteristics, including the types of sedation, sedation protocols, and clinical outcomes, were extracted. The Preferred Reporting Items for Systematic Reviews and Meta-Analyses (PRISMA) reporting guideline was followed. A random-effects model was used to pool results from articles for the meta-analysis.

**Main Outcomes and Measures:**

The primary outcomes of interest were duration of MV and length of PICU stay. Secondary outcomes included total sedative dose requirement, adverse events (eg, complications associated with MV, withdrawal, and delirium), and mortality.

**Results:**

A total of 6 RCTs with 2810 pediatric patients (1569 males [55.8%]; mean age, 26.5 [95% CI, 15.0-37.9] months) were included in the final analysis; patients had a mean PRISM (Pediatric Risk of Mortality) score of 13.68 (95% CI, 10.75-16.61). Compared with continuous IV sedation, DSI was associated with a reduction in length of PICU stay (5 studies, n = 2770; mean difference [MD], −1.45 [95% CI, −2.75 to −0.15] days; *P* = .03]. There was no difference in MV duration (5 studies, n = 2750; MD, −0.93 [95% CI, −1.89 to 0.04] days; *P* = .06), total doses of midazolam (3 studies, n = 191; MD, −1.66 [95% CI, −3.95 to 0.63] mg/kg) and morphine used (2 studies, n = 189; MD, −2.63 [95% CI, −7.01 to 1.75] mg/kg), or adverse events (risk ratio [RR], 1.03 [95% CI, 0.74-1.42]; *P* = .88). There was no difference in mortality between patients exposed vs not exposed to DSI (RR, 0.89 [95% CI, 0.55-1.46]; *P* = .65).

**Conclusions and Relevance:**

This systematic review and meta-analysis found that use of DSI in pediatric patients was associated with reduced length of PICU stay with no increase in adverse events. Further research is needed to ascertain whether this strategy is associated with improved neurodevelopmental outcomes in PICU survivors.

## Key Points

**Question:**

Does daily sedation interruption (DSI) carry any benefit over continuous intravenous sedation in patients receiving invasive mechanical ventilation (MV) in the pediatric intensive care unit (PICU)?

**Findings:**

In this systematic review and meta-analysis of 6 randomized clinical trials that included 2810 pediatric patients, DSI use was associated with reduced length of PICU stay, with no difference from continuous sedation in duration of MV, total sedative dosing, and adverse events.

**Meaning:**

Findings of this study suggest that further research is needed to ascertain whether DSI is associated with improved neurodevelopmental outcomes in PICU survivors.

## Teaser

This systematic review and meta-analysis evaluates the clinical outcomes associated with daily sedation interruption vs continuous intravenous sedation in children receiving mechanical ventilation support.

## Introduction

Sedation plays a paramount role in a critically ill pediatric patient who is supported by invasive mechanical ventilation (MV) to ease anxiety, augment and prioritize comfort, enable the performance of invasive procedures, reduce metabolic demands, facilitate neuroprotection, and allow better synchronization with the ventilator.^1,2,3^ The goals of sedation are to be individualized and titrated according to the underlying pathology, with regular sedation assessments using validated tools (eg, Face, Legs, Activity, Cry, and Consolability scale; Ramsay Sedation Scale and Richmond Agitation Sedation Scale; COMFORT Scale or COMFORT Behavior Scale; and State Behavioral Scale for sedation) to ensure patients are optimally sedated when under MV support.^2^ Targeting optimal levels of sedation to facilitate spontaneous breathing, prevent delirium, and minimize withdrawal risk and early mobilization is part of the holistic management of a mechanically ventilated, critically ill pediatric patient.^4,5^

Titration to achieve the optimal depth of sedation to target the aforementioned goals while avoiding oversedation to minimize its adverse outcomes can be challenging due to interpatient variability affected by disease trajectory and evolution of disease.^6^ Associated short-term harm of continuous intravenous (IV) sedation in both undersedated or oversedated pediatric patients ranges from tolerance, withdrawal due to reduced kidney and hepatic metabolism, and morbidities such as prolonged MV and hospital stay.^7^ Neurotoxic properties of sedation in neuronal cells coupled with its interaction with critical illness brain stress and injury are potentially associated with long-term harm to the developing brains of children and adolescents.^8^ The search for the optimal sedation strategy in pediatric patients continues amid better outcomes, such as reduction in duration of MV and intensive care unit (ICU) stay, associated with the use of daily interrupted sedation practices in adult ICUs.^9,10,11^ However, insufficient evidence exists at present to allow for the direct extrapolation of adult data to pediatric practice.^12^

One such strategy to reduce excessive sedation exposure and its adverse outcomes is the use of daily sedation interruption (DSI), which refers to a temporary interruption of sedation,^13^ in contrast to continuous administration. As most existing randomized clinical trials (RCTs) on sedation were based on adult populations,^13,14,15^ little is known about the effectiveness of DSI in pediatric patients with regard to duration of MV, weaning off sedation, and incidence of adverse events (eg, agitation and accidental extubation).

This systematic review and meta-analysis updates an analysis of the use of DSI in pediatric patients, addressing the limitations of the previous systematic review and meta-analysis^12^ done in 2018. Using current medical literature, the present analysis aimed to compare the clinical outcomes of DSI vs continuous IV sedation in patients receiving MV support in the pediatric ICU (PICU).

## Methods

### Search Strategy and Study Eligibility

With critical input from an experienced medical librarian (R.C.), we conducted a systematic search for studies using a combination of predefined keywords and Medical Subject Headings (*pediatric intensive care unit*, *daily sedation interruption*, *continuous sedation infusion*, and *sedation protocol*) in 5 major databases: PubMed, Embase, Web of Science, CINAHL (Cumulated Index to Nursing and Allied Health Literature), and Cochrane Central Register of Controlled Trials from the time of database inception through October 31, 2023. We defined DSI as any temporary cessation in sedation and continuous IV sedation as the administration of sedative agents without interruption, which can include protocolized sedation with lighter sedation targets.^13^ There was no language restriction in the search strategy. This systematic review and meta-analysis was registered in PROSPERO (CRD42022314028). We followed the Preferred Reporting Items for Systematic Reviews and Meta-Analyses (PRISMA) reporting guideline.^16^

The primary outcomes of interest were duration of MV and length of PICU stay. Secondary outcomes were mortality (all types of mortality rate measurements reported were accepted, such as PICU, hospital, and 28-day mortality), total sedative dose requirement (defined as total amount of sedative used during the PICU admission, standardized to a common unit [milligrams per kilogram] as appropriate to the sedative used), duration of sedation weaning (defined as duration in hours from the time a patient was deemed by the medical team to be fit for weaning off sedation to the time all sedation was stopped), and adverse events (eg, complications related to MV, withdrawal, and delirium).

Studies were included if the study cohort was aged 18 years or younger, required sedation for MV in the PICU, and reported at least 1 of the primary outcomes of duration of MV and/or length of PICU stay. We included systematic reviews, RCTs, and observational studies (both retrospective and prospective). Studies with a subpopulation who fulfilled the criteria were included if the data subset could be extracted. Studies from prospective databases, post hoc analyses of RCTs, or studies with a retrospective or prospective arm were also included as long as the study population fulfilled the criteria. Exclusion criteria were studies without a clear study design; studies conducted exclusively in the neonatal ICU, in the adult ICU, or with patients who received surgical anesthesia; conference abstracts or narrative reviews; and studies involving nonventilated patients requiring sedation in the PICU.

### Data Extraction and Quality Assessment

Two of us (P.R.R. and K.H.Y.H.) screened titles and abstracts for eligibility and retrieved full-text articles for a thorough examination of their eligibility. Any disagreements were resolved by discussion and, if needed, adjudicated by an independent third party (T.S.W.T.). Data were collected from eligible studies by 2 of us (P.R.R. and K.H.Y.H.) using a standard data collection form. Data collected included author names and year of publication, clinical variables (including age, sex, presence of comorbidities or congenital defects, severity scores, PICU support modalities, and PICU admission diagnosis), description of DSI or continuous IV sedation drug used, sedation scores, sedation protocol, mean sedative doses used, duration of MV, length of PICU stay, duration of sedation weaning and mortality rate reporting, and any adverse events and neurological cognitive outcomes.

If required, missing data from the included studies were requested from the respective authors. All results were tracked using the Covidence platform (Covidence). Assessment of the methodological quality of the studies was carried out using the *Cochrane Handbook for Systematic Reviews of Interventions*^17^ for RCTs and the Newcastle-Ottawa Scale^18^ for observational studies.

### Statistical Analysis

A meta-analysis of all included articles was conducted using R, version 4.2.2 (R Project for Statistical Computing). We used a random-effects model to pool results from articles, accounting for variation between studies. The primary outcomes (ie, length of PICU stay and duration of MV) were treated as continuous data. Secondary outcomes of adverse events and sedative dosing were treated as binary and continuous data, respectively. Continuous outcomes were pooled using the mean difference (MD) or standardized MD, whichever was applicable; adverse events were pooled using risk ratios (RRs). All outcomes were reported with a 95% CI. Potential publication bias was presented in a funnel plot. If a study reported a median with IQR, the mean and SD were estimated by taking into account the sample size and smoothly changing weight in the estimation.^19^ Two-sided *P* < .05 was considered statistically significant. Heterogeneity was evaluated using the χ^2^-based q test and *I*^2^ test.^20^ A q value less than 0.05 and *I*^2^ greater than 50% indicated the possibility of significant heterogeneity.^21^

To address heterogeneity, subgroup analysis comparing medical vs surgical cohorts was performed. Additionally, the 7 domains of potential risk of bias were analyzed across all included RCTs.

## Results

### Study and Patient Characteristics

**Figure 1.**
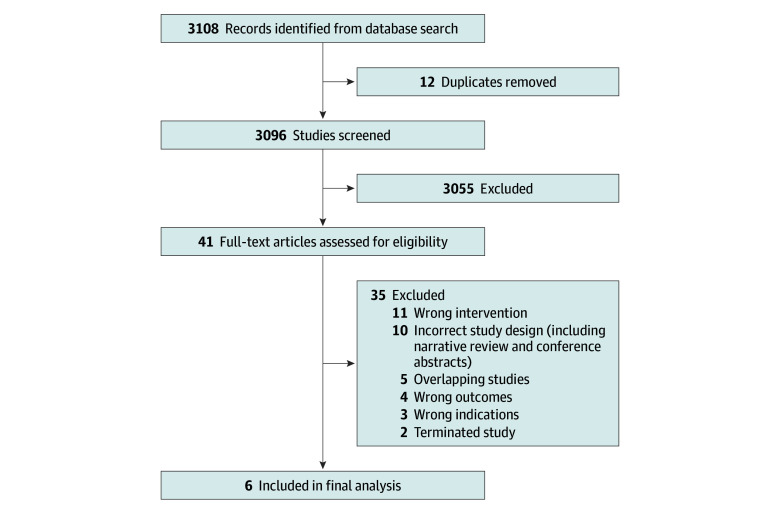
Systematic Review Study Selection PRISMA Flowchart

A total of 2810 pediatric patients were identified from the 6 RCTs^7,9,22,23,24,25^ that we deemed eligible from the 3108 references retrieved from the database search (Figure 1). These patients were included in the qualitative analysis, comprised 1569 males (55.8%) and 1241 females (44.2%), had a mean age of 26.5 (95% CI, 15.0-37.9) months, and had a mean PRISM (Pediatric Risk of Mortality) score of 13.68 (95% CI, 10.75-16.61). The median (range) number of participants per study was 60 (30-2449). Eighty patients (2.8%) were postsurgical cases.

After screening the titles and abstracts, we examined 41 full-text articles for eligibility. The 6 RCTs^7,9,22,23,24,25^ we included in the final qualitative and quantitative analyses were conducted between 2012 and 2018, of which 2 were multicenter studies^7,22^ and 4 were single-center studies.^9,23,24,25^ Five of the 6 studies^7,9,22,24,25^ were conducted in a mixed PICU, while 1 study^23^ was in a cardiac-surgical PICU. Two RCTs were conducted in Asia (India and Indonesia; n = 142), 2 in Europe (the Netherlands; n = 159), and 2 in North America (US; n = 2509) (Table). There was blinding in 3 RCTs,^7,9,23^ while 2 were nonblinded studies.^22,25^ All except 1 study^24^ included both midazolam and morphine as first-line medications in the protocol, with variations in dosing regimens and allowances for alternatives, such as fentanyl, in the event of hemodynamic instability or reactive airway disease.^22^ In 3 of 6 RCTs,^7,22,23^ the sedation protocol was nurse driven with physician oversight.

**Table.** Characteristics of the 6 Included Randomized Clinical Trials

Source, publication year	Country, region	Study period	Outcomes	Patient population (sample size)	Inclusion criteria	Exclusion criteria	Control group	Intervention group
Gupta et al,^25^ 2012	Single center; India, Asia	January to December 2007	Primary: To determine the total duration of MV and PICU stay Secondary: To determine the number and percentage of days awake on sedative infusions, frequency of adverse events, and total dose of sedatives with equivalent cost	Patients admitted to PICU in a tertiary care teaching and referral hospital receiving MV for >48 h (n = 102)	All patients admitted to PICU requiring MV for >48 h	Patients requiring PIP>28 mm Hg	Continuous IV sedation infusion with midazolam and morphine	Sedation interruption protocol with DSI at 8 am and restarted at 50% of previous dose if patient became fully awake or agitated
Verlaat et al,^9^ 2014	Single center; the Netherlands, Europe	November 2004 to October 2006	Primary: To determine the feasibility of DSI in critically unwell patients and its effects on total amount of sedatives used Secondary: Nil	Patients from mixed medical and surgical PICUs (n = 30)	Age 0-12 y; intubated and received MV for 24 h and expected to need it for >48 h at time of inclusion	High PIP; safety flagged by medical staff (fear of accidental extubation in difficult airway, hemodynamic instability); no informed consent	Continuous infusion with midazolam and morphine (dose based on clinical judgment)	Continuous sedation infusions stopped daily at 1 pm (quiet part of the day with minimal activity) and restarted if COMFORT Behavior scale score of ≥17
Curley et al,^22^ 2015	Multicenter; US, North America	June 2009 to December 2013	Primary: To determine whether critically unwell patients managed with a nurse-implemented and goal-directed sedation protocol would require a lesser duration of MV than patients receiving routine care Secondary: Nil	Patients from 31 PICUs recruited from PALISI Network (n = 2449)	Age 2-17 y; ventilated for acute airway disease or lung parenchymal disease; PICUs without existing sedation protocol, agreeable for study, and good leadership among physicians and nurses	Length of MV unlikely to be affected by sedation (eg, postoperative); extubated within 24 h	Continuous IV sedation infusion based on standard institutional practice (without protocol)	Sedation interruption protocol based on the RESTORE protocol
Vet et al,^7^ 2016	Multicenter; the Netherlands, Europe	October 2009 to August 2014	Primary: To compare ventilator-free days in those with DSI combined with PS vs PS alone Secondary: To compare short-term health-related QOL and PTSD symptoms between DSI with PS vs PS alone	Patients from 3 tertiary medical-surgical PICUs (n = 129)	Age 0-18 y; at least PMA of ≥37 wks; requiring MV for at least 48 h; need for sedative medications	Anticipated death or withdrawal of life support within 48 h; inability to assess sedation due to underlying neurologic comorbidity; cardiac, respiratory, or neurological instability that may not withstand insufficient sedation; therapeutic hypothermia after CPR; challenging airway; planned duration of intubation for procedure; admission for ECMO; previously ventilated/sedated >48 h from a transferring PICU; no informed consent	Protocolized sedation based on COMFORT Behavior scale score (>22 undersedation; <11 oversedation), if sedation required midazolam started and titrated up to 0.3 mg/kg/h; if required, morphine added (up to 0.03 mg/kg/h)	If the patient passed safety screen after 24 h, then all continuous infusion converted to blinded saline infusions; if required (patient uncomfortable or cardiorespiratory instability), then load with midazolam 0.1 mg/kg bolus, then sedative infusions restarted at 50% of previous dose and titrated according to sedation protocol
Azis et al,^24^ 2016	Single center; Indonesia, Asia	March to May 2015	Primary: To measure total duration of MV Secondary: To identify time for patients to awaken on MV and frequency of adverse events	Patients receiving MV in PICU (n = 40)	Age 1-18 y; ventilated for at least 24 h	Anticipated death within 24 h; withdrawal of life support; neurologic comorbidity affecting ability to score sedation; received CPR; need continuous sedative infusions for seizures; admitted to PICU after previous MV or use of sedative drugs	Continuous IV sedation infusion with midazolam titrated to achieve a COMFORT Pain scale score 11-22, with interruption only done 4-6 h before intended weaning from MV	Sedation interruption protocol with sedative infusion discontinued after first 24 h on MV, with infusion restarted if patient was uncomfortable or agitated as per COMFORT score
Penk et al,^23^ 2018	Single center; US, North America	August 2014 to May 2016	Primary: To assess degree of comfort achieved in patients receiving intermittent vs continuous and intermittent dosing when adjunct medications were used Secondary: Nil	Patients scheduled for cardiac surgery (n = 60)	Age 3 mo to 4 y; early extubation within 3 h of admission to cardiac ICU; with a midline sternotomy	Kidney disease; bleeding diathesis or previous GI bleed within 2 mo; chronic liver disease or ALT>300 U/L at preoperative visit; developmental delay impeding sedation scoring; ≥3 sternotomies; admission to PICU	Continuous morphine and midazolam infusion after extubation, with intermittent open-label morphine or midazolam boluses if FLACC scale score of ≥4 for 24 h; then transitioned to standard care (convert to oral medications)	Intermittent open-label midazolam/morphine boluses if FLACC scale score of ≥4, with continuous normal saline infusion for 24 h; then transitioned to standard care (convert to oral medications)

Abbreviations: ALT, alanine aminotransferase; CPR, cardiopulmonary resuscitation; DSI, daily sedation interruption; ECMO, extracorporeal membrane oxygenation; FLACC scale, Face, Legs, Activity, Cry and Consolability; GI, gastrointestinal; ICU, intensive care unit; IV, intravenous; MV, mechanical ventilation; PALISI, Pediatric Acute Lung Injury and Sepsis Investigators Network; PICU, pediatric intensive care unit; PIP, peak inspiratory pressure; PMA, postmenstrual age; PS, protocolized sedation; PTSD, posttraumatic stress disorder; QOL, quality of life.

SI conversion factor: To convert ALT to microkatals per liter, multiply by 0.0167.

Study characteristics, including inclusion and exclusion criteria, are summarized in the Table. Patient characteristics, sedation regimens and dosing, and outcomes are summarized in eTables 1 to 3 in Supplement 1.

The most common medical indications requiring admission to the PICU for MV had respiratory etiologies (2323 [82.7%]), including acute respiratory distress syndrome, bronchiolitis, pneumonia, and status asthmaticus.^7,9,22^ Other medical indications included cardiomyopathy and congenital heart disease, septic shock, and neurological reasons.^7,9,24,25^ The most common surgical indications requiring admission to the PICU were postoperative cardiac surgical procedures, ranging from atrial septal defect or ventricular septal defects to repair of total anomalous pulmonary venous drainage or tetralogy of Fallot.^23^

Midazolam was the most common sedative agent assessed across all RCTs, followed by morphine in 5 RCTs.^7,9,22,23,25^ Clonidine, ketamine, and fentanyl were examined in 2 RCTs.^7,22^ Dexmedetomidine and lorazepam were evaluated in 1 RCT^22^ and propofol in another RCT.^7^ Dosing regimens for the sedatives and protocols used for DSI varied across the trials according to their respective institutional practices. Study methodologies are described in eTable 2 in Supplement 1.

### Association of DSI With MV Duration and Length of PICU Stay

**Figure 2.**
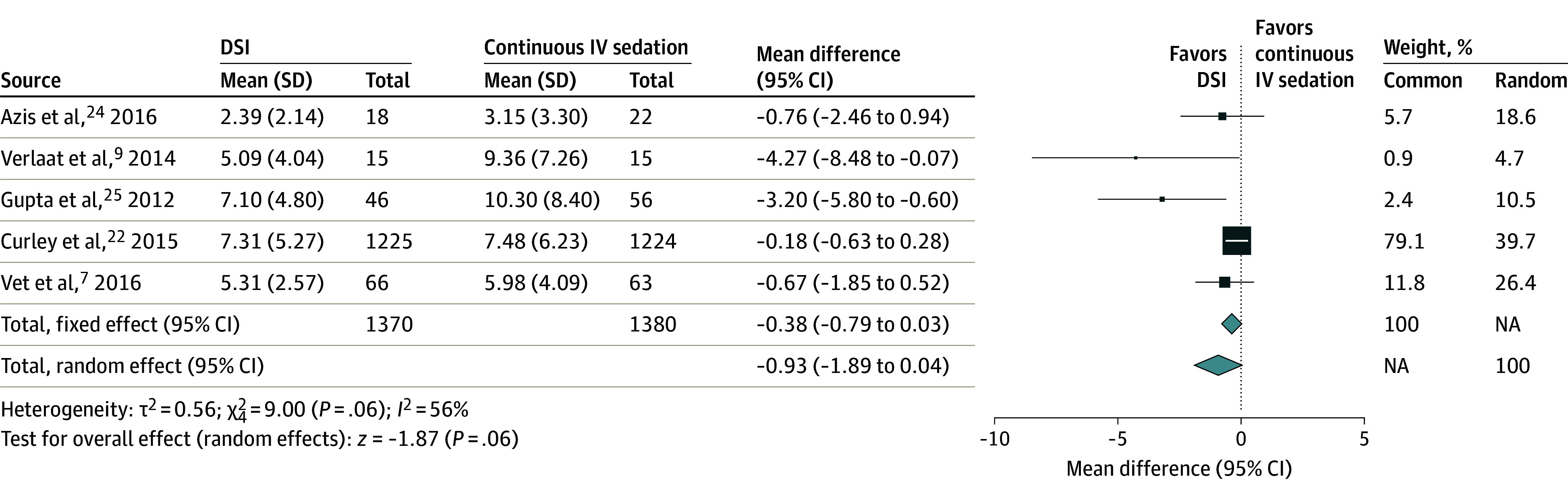
Duration of Mechanical Ventilation in Daily Sedation Interruption (DSI) vs Continuous Intravenous (IV) Sedation Square data marker sizes represent the weight of the study; error bars indicate 95% CIs. NA indicates not applicable.

**Figure 3.**
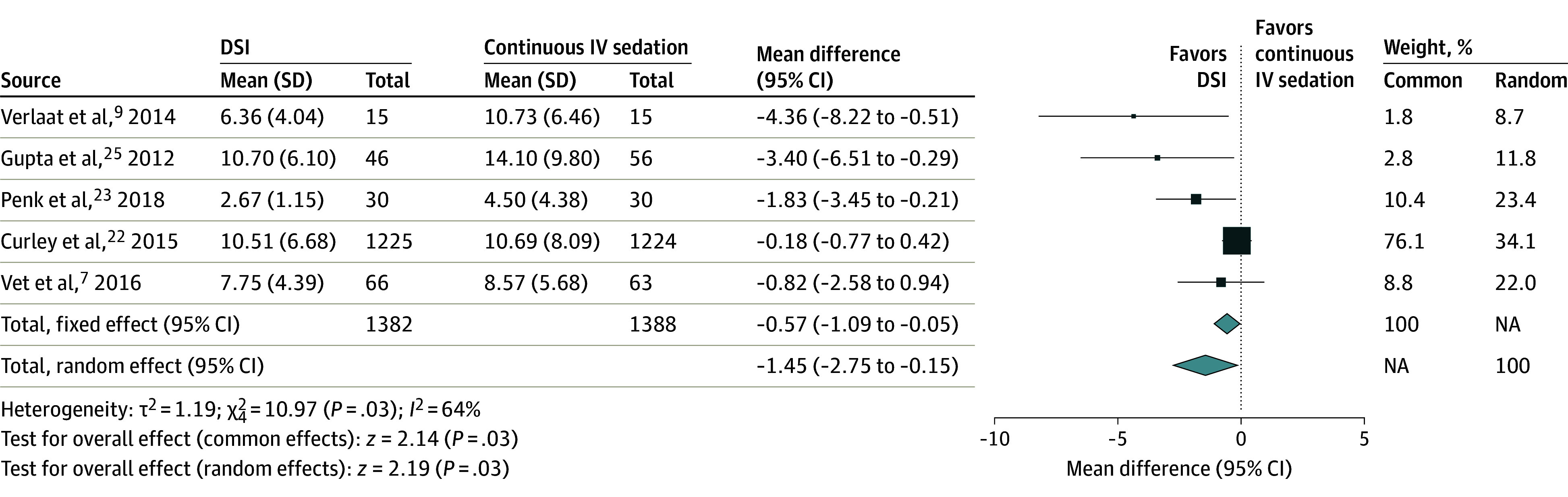
Length of Intensive Care Unit Stay in Daily Sedation Interruption (DSI) vs Continuous Intravenous (IV) Sedation Square data marker sizes represent the weight of the study; error bars indicate 95% CIs. NA indicates not applicable.

Five RCTs reported the primary outcomes of duration of MV^7,9,22,24,25^ and length of PICU stay.^7,9,22,23,25^ Patients in the DSI group had a shorter duration of MV compared with patients in the continuous IV sedation group (5 studies,^7,9,22,24,25^ n = 2750; MD, −0.93 [95% CI, −1.89 to 0.04] days), although the results were not significant (*P* = .06) (Figure 2). Meta-analysis of these 5 studies^7,9,22,23,25^ revealed that the DSI group had a shorter length of PICU stay compared with the continuous IV sedation group (n = 2770; MD, −1.45 [95% CI, −2.75 to −0.15] days), and these results were significant (*P* = .03) (Figure 3). One RCT^24^ was excluded due to incomplete data for the DSI group and no data reported for the control group.

### Association of DSI With Sedative Dosing and Adverse Events

Secondary outcomes were similar in both DSI and continuous IV sedation groups (eFigures 1 and 2 in Supplement 1), with no significant difference detected in total doses of midazolam (3 studies,^7,23,25^ n = 191; MD, −1.66 [95% CI, −3.95 to 0.63] mg/kg) and morphine (2 studies,^7,23^ n = 189; MD, −2.63 [95% CI, −7.01 to 1.75] mg/kg). Both groups had similar adverse event profiles (5 studies,^7,9,22,24,25^ n = 2750; RR, 1.03 [95% CI, 0.74-1.42]; *P* = .88), with no difference in mortality (4 studies,^7,9,22,25^ n = 2710; RR, 0.89 [95% CI, 0.55-1.46]; *P* = .65). None of the RCTs reported sedation-free days as an outcome.

The most commonly reported adverse event in the continuous IV sedation group vs the DSI group was accidental extubation (0.6% vs 0.4%) in 5 RCTs.^7,9,22,24,25^ Other adverse events reported in the continuous IV sedation group vs the DSI group were pneumothorax (0.5% vs 0.4%), inadequate analgesia (12.4% vs 13.9%) vs oversedation (0.1% vs 0%), withdrawal (8.1% vs 10.6%), hemodynamic instability (0.1% vs 0.1%), ventilation-associated complications, such as postextubation stridor (3.9% vs 6.3%), and immobility-related complications such as ulcers (1.4% vs 0.4%) (eTable 3 in Supplement 1). No adverse events were noted in 1 RCT,^23^ and mortality data were not reported in 2 RCTs.^23,24^ Delirium was not described as an outcome of interest in all included studies. One RCT^22^ reported that delirium could not be assessed because pediatric assessment tools were unavailable on commencement of the trial.

### Subgroup Analysis

Duration of length of PICU stay was compared between medical and surgical cohorts to ascertain whether DSI had a comparable outcome between both cohorts. In the medical cohort (2 studies,^22,25^ n = 2521; MD, −1.25 [95% CI, −4.23 to 1.73]), there was no evidence of a significant difference between the continuous IV sedation and DSI groups. In the surgical cohort (1 study,^23^ n = 60; MD, −1.83 [95% CI, −3.45 to −0.21]), the DSI group had a reduced duration of PICU stay (eFigure 3 in Supplement 1). Two RCTs^7,9^ were not included due to mixed data between medical and surgical cohorts, and data were not available for 1 RCT^24^ with a purely medical cohort. Subgroup analysis for the duration of MV was not carried out due to incomplete data from the 1 RCT^23^ with a purely surgical cohort.

### Risk of Bias 

**Figure 4.**
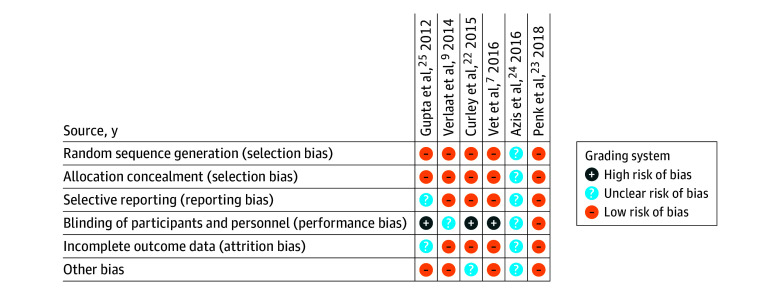
Risk of Bias Across All Included Studies as Assessed by Independent Reviewers

Selection and reporting biases were low in most of the 6 RCTs, although performance and detection biases were higher because blinding was not standard across all study methodologies (Figure 4). Attrition bias and other biases were also low across the studies. The number of patients lost to follow-up was assumed to be minimal or 0 given that the patients were followed up until time of discharge from the PICU or the hospital. One RCT^9^ followed up the cohort for 1 year after study enrollment. Two RCTs^24,25^ did not provide data on patients lost to follow-up. The asymmetric funnel plot raised concerns for a potential publication bias likely attributed to study heterogeneity (eFigure 4 in Supplement 1).

## Discussion

Since the 2018 systematic review and meta-analysis on DSI in pediatric patients,^12^ there have been considerable advancements in the provision of sedation in critically ill patients. The present systematic review and meta-analysis found that DSI was associated with reduced length of PICU stay in patients who received DSI. There was no significant difference in MV duration, total doses of midazolam and morphine used, or adverse events between patients in the DSI and continuous IV sedation groups.

One of the proposed benefits of DSI is a lower risk of oversedation and, in turn, lower risk of remaining intubated and ventilated for a prolonged period, which has inherent complications such as ventilator-associated pneumonia.^3^ This benefit was observed in the previous systematic review and meta-analysis on pediatric DSI,^12^ which was consistent with findings in a systematic review on adult DSI^3^ but was not consistent across other adult studies.^26,27^ The discrepancy within the findings could be attributed to study heterogeneity, as ventilatory requirements may vary across different disease pathologies as well as different MV weaning strategies across different studies. In addition, disease pathologies requiring invasive therapies (eg, extracorporeal membrane oxygenation or continuous kidney replacement therapy) or surgical conditions (eg, postoperative neurosurgical interventions or cardiac surgical procedures requiring deeper sedation or delayed weaning to mitigate withdrawal risk) may inevitably prolong MV duration in pediatric patients.^28^ However, certain cohorts, such as postsurgical patients, once extubated and off sedation, may experience shorter overall length of PICU stay. Similar heterogeneity was seen across the RCTs as evidenced by differences in target populations, which will incontrovertibly confound the outcomes of DSI and interpretation of its perceived benefits. However, we used a random-effects model in an attempt to account for effect heterogeneity because the true effect size may not be same across all studies.

While there is a risk of insufficient sedation leading to agitation secondary to pain and, in turn, adverse outcomes such as accidental extubation,^29^ DSI potentially enables patients to wean off their sedation faster without worrying about drug bioaccumulation, get extubated sooner, and thus leave the PICU earlier.^30^ In the RCTs analyzed, reduced duration of PICU stay in the DSI cohort was similarly observed by Verlaat et al^9^ and Gupta et al.^25^ This finding was also noted in a systematic review of 45 RCTs with 5493 adults admitted to the ICU whereby DSI was associated with shorter overall length of ICU stay.^3^ However, this was not a consistent finding in other systematic reviews involving adult patients.^13,26^ One can posit that confounding factors, such as the course of an illness and associated complications, a poor premorbid state or other comorbidities, and greater severity of illness in terms of higher mortality scores, can also contribute to an extended ICU stay, which may not always be directly affected by sedation practices.^31^

Although we found that the DSI group had a shorter length of PICU stay, it did not correspond to a significantly shortened duration of MV; however, there was still a reduction of 0.93 days compared with the continuous IV sedation group. One may expect that a shorter PICU stay would go in tandem with a shorter duration of MV. A finding from this study was similar to that noted in another systematic review^32^ comparing outcomes of duration of MV, length of ICU stay, and hospital mortality between pediatric and adult patients in DSI vs continuous IV sedation groups: overall, the duration of MV, length of ICU stay, and total sedative dose were not reduced even in the DSI cohort because of the heterogeneity of the studies included. We postulated that this observation can be attributed to institution-dependent sedation weaning strategies to prevent complications (eg, withdrawal or delirium), illness trajectory or postsurgical course, elective admissions to the PICU, and difference in the proportion of patients with chronic illness. However, it is plausible that patients could have stayed in the PICU for other sedation sequelae, such as delirium, which were not reported in the RCTs.

The present examination of all RCTs on this topic to date showed that while there was no difference in duration of MV or sedative dosing, there was no difference in mortality outcome, and the overall length of PICU stay was reduced with no difference in adverse outcomes, suggesting that less sedation (DSI) may be as safe or safer than more sedation (continuous IV infusion). This is contrary to a 2022 Italian consensus guideline on analgosedation in patients admitted to the PICU,^30^ which cautioned against the use of DSI in view of conflicting RCT findings that DSI was not associated with reduced duration of MV, length of ICU stay, and amount of sedation but was associated with increased mortality. Nonetheless, the authors of the Italian guidelines also reinforced that implementation of a DSI protocol should not be the only factor in improving overall quality of care but should be part of a framework that includes educating the health care team and empowering nurses regarding reducing unnecessary sedation for better outcomes of intubated and ventilated patients in the PICU.^33^ This finding was also echoed in a review of sedation protocols in the ICU by Balit et al,^34^ which showed that while there was no difference in the duration of MV or length of PICU stay, a sedation protocol helped with keeping patients calmer when awake as well as improving overall interprofessional communication by establishing clearer goals of care to maintain sedation targets with regular sedation assessments. We believe that a DSI protocol alone may not change overall outcomes, but it would play a role in improvement of outcomes. We recommend that future studies compare important clinical outcomes before and after implementation of sedation protocols, with DSI as part of the care bundles in PICUs.

We did not find evidence of a difference in adverse events between the DSI and continuous IV sedation groups, contrary to the previous systematic review and meta-analysis,^12^ which reported a slightly higher rate of adverse events in the DSI group. While 1 RCT^22^ reported significant adverse events, the other 6 studies did not find many, which reflects the lower risk profile of DSI in pediatric patients. Accidental extubation, withdrawal and need for restraints, and inadequate pain control are all possible risks, but the managing medical and nursing teams should tailor the DSI regimen on a case-by-case basis. It would be appropriate to start this practice in patients with a lower risk profile rather than in patients with a higher risk profile, such as those with a difficult airway. Moreover, there are possible confounders contributing to an increased risk of adverse events, such as depth of sedation, nursing manpower, and, in turn, quality of nursing care rendered to each patient.^35^

This study also did not show a significant difference in sedative dosing. In contrast, the review by Balit et al^34^ showed a favorable outcome from sedation protocols associated with reduced midazolam dose without corresponding harm to patients in terms of adverse events and ability to meet sedation targets. There is growing evidence^30,36,37^ that midazolam has adverse implications for neurocognitive development and has a dose-dependent association with delirium. Further longitudinal studies are needed to identify long-term psychological outcomes, such as posttraumatic stress disorder, anxiety, and depression.^38^ While studies have shown no harm associated with transient anesthesia use in pediatric patients, a study on patients aged 0 to 8 years with acute respiratory failure requiring PICU admission and subjected to PICU interventions and sedation for many days reported that these patients had an overall lower intelligence quotient compared with well siblings within the same household.^39^ Hence, long-term neurodevelopmental outcomes remain important considerations for an intensivist managing critically unwell pediatric patients.

### Limitations

The findings of this systematic review and meta-analysis should be interpreted within the context of its limitations. Similar to prior reviews, the heterogeneity of studies remains a limitation in the current review.^12,13^ The heterogeneity of studies can be explained by the heterogeneity of clinical practices^26^ across different PICUs (cardiac, surgical, and medical), which may indirectly affect the outcomes of pediatric patients receiving DSI. The majority of patients included had medical diagnoses, and the findings may not be applicable to surgical patients. Larger trials focusing on surgical patients should be considered. Sample sizes of the majority of the included RCTs were also small, which would reduce the power of this study and hence the generalizability of the conclusion. However, the small samples reiterate the dearth of data available for conducting a large RCT before DSI can be recognized as a safe and acceptable sedation strategy for the pediatric population. Most of the RCTs focused on midazolam and morphine; the outcome of DSI using different sedatives and different combinations remains to be fully investigated. While DSI has been shown not to have adverse psychological outcomes in adults,^40^ the present study did not examine the long-term psychological or neurocognitive outcomes in pediatric patients receiving DSI.

Moreover, limited data across the 6 RCTs did not allow us to perform meaningful subgroup analyses of the differences in primary outcomes between cohorts with varying medical diagnoses (respiratory vs neurologic) or comorbidities (neurologic vs non-neurologic). Data (eg, assessment for delirium, sedation-free days, and ventilator-free days) that may explain the lack of a detectable difference in sedation burden, with more complete information on adverse events, were also not available.

## Conclusions

In this systematic review and meta-analysis, use of DSI was associated with shorter length of PICU stay without increased adverse events but was not associated with a reduction in the duration of MV and sedative dosing. While this conclusion may be of uncertain clinical significance in view of the limitations of existing systematic reviews on this divisive topic, larger-scale studies delineating the safety profile of DSI in critically ill pediatric patients receiving MV support are needed to strengthen these findings. Additionally, future studies should investigate the association of DSI with improved neurodevelopmental outcomes in PICU survivors.

## Notes

Supplement 1. **eFigure 1.** Sedation Dosing in Daily Sedation Interruption (DSI) vs Continuous Intravenous (IV) Sedation **eFigure 2.** Adverse Events in Daily Sedation Interruption (DSI) vs Continuous Intravenous (IV) Sedation **eFigure 3.** Subgroup Analysis of Surgical vs Medical Cohort of Patients Receiving Daily Sedation Interruption (DSI) vs Continuous Intravenous (IV) Sedation **eFigure 4.** Funnel Plot for Length of Stay in PICU **eTable 1.** Characteristics of Patients Included in Studies **eTable 2.** Daily Sedation Interruption (DSI) vs Continuous Intravenous (IV) Sedation by Dosing Regimen or by Distribution Across Different Sedative Agents **eTable 3.** Patient Outcomes in Both Daily Sedation Interruption (DSI) and Continuous Intravenous (IV) Sedation Groups

Supplement 2.
Data Sharing Statement
