# Supplementary material for: Daily Sedation Interruption vs Continuous Sedation in Pediatric Patients Receiving Mechanical Ventilation: A Systematic Review and Meta-analysis
Source: JAMA Netw Open. 2024 Aug 7;7(8):e2426225. doi: 10.1001/jamanetworkopen.2024.26225 (PMC11307139; doi:10.1001/jamanetworkopen.2024.26225)
Supplement: Supplement 1. — eFigure 1. Sedation Dosing in Daily Sedation Interruption (DSI) vs Continuous Intravenous (IV) Sedation eFigure 2. Adverse Events in Daily Sedation Interruption (DSI) vs Continuous Intravenous (IV) Sedation eFigure 3. Subgroup Analysis of Surgical vs Medical Cohort of Patients Receiving Daily Sedation Interruption (DSI) vs Continuous Intravenous (IV) Sedation eFigure 4. Funnel Plot for Length of Stay in PICU eTable 1. Characteristics of Patients Included in Studies eTable 2. Daily Sedation Interruption (DSI) vs Continuous Intravenous (IV) Sedation by Dosing Regimen or by Distribution Across Different Sedative Agents eTable 3. Patient Outcomes in Both Daily Sedation Interruption (DSI) and Continuous Intravenous (IV) Sedation Groups [file jamanetwopen-e2426225-s001.pdf]

Supplemental Online Content

Shu Wen Toh T, RR P, Ho KHY, et al. Daily sedation interruption vs continuous sedation in pediatric patients receiving mechanical ventilation: a systematic review and meta-analysis. *JAMA Netw Open*. 2024;7(8):e2426225.
doi:10.1001/jamanetworkopen.2024.26225

eFigure 1. Sedation Dosing in Daily Sedation Interruption (DSI) vs Continuous Intravenous (IV) Sedation

eFigure 2. Adverse Events in Daily Sedation Interruption (DSI) vs Continuous Intravenous (IV) Sedation

eFigure 3. Subgroup Analysis of Surgical vs Medical Cohort of Patients Receiving Daily Sedation Interruption (DSI) vs Continuous Intravenous (IV) Sedation

eFigure 4. Funnel Plot for Length of Stay in PICU

eTable 1. Characteristics of Patients Included in Studies

eTable 2. Daily Sedation Interruption (DSI) vs Continuous Intravenous (IV) Sedation by Dosing Regimen or by Distribution Across Different Sedative Agents

eTable 3. Patient Outcomes in Both Daily Sedation Interruption (DSI) and Continuous Intravenous (IV) Sedation Groups

This supplemental material has been provided by the authors to give readers additional information about their work.

© 2024 Shu Wen Toh T et al. *JAMA Network Open*

eFigure 1. Sedation dosing in daily sedation interruption versus continuous intravenous sedation

Study or Subgroup	DSI Mean	DSI SD	DSI Total	Continuous IV sedation Mean	Continuous IV sedation SD	Continuous IV sedation Total	Weight (common)	Weight (random)	Std. Mean Difference IV, Fixed + Random, 95% CI
**Sedation = Midaz**									
Jamie S Penk et al, 2018	0.18	0.18	30	0.90	0.17	30	1.9%	8.6%	−4.06 [−4.96; −3.15]
Nienke J. Vet et al, 2015	14.81	11.35	66	21.94	23.94	63	12.6%	9.2%	−0.38 [−0.73; −0.03]
Nienke J. Vet et al, 2016	14.38	10.60	32	21.21	26.16	32	6.3%	9.1%	−0.34 [−0.83; 0.16]
Gupta et al., 2012	7.10	4.70	46	11.00	6.90	56	9.5%	9.2%	−0.64 [−1.04; −0.24]
**Total (common effect, 95% CI)**			**174**			**181**	**30.3%**	**--**	**−0.68 [−0.91; −0.46]**
**Total (random effect, 95% CI)**							**--**	**36.2%**	**−1.32 [−3.04; 0.40]**
Heterogeneity: $Tau^2 = 3.0068$; $Chi^2 = 58.27$, df = 3 ($P < 0.01$); $I^2 = 95\%$									
**Sedation = Morphine**									
Jamie S Penk et al, 2018	0.23	0.14	30	0.90	0.13	30	1.4%	8.4%	−4.90 [−5.93; −3.86]
Nienke J. Vet et al, 2015	1.03	0.73	66	1.58	1.67	63	12.5%	9.2%	−0.43 [−0.77; −0.08]
Nienke J. Vet et al, 2016	0.97	0.60	32	1.72	2.00	32	6.2%	9.1%	−0.50 [−1.00; −0.00]
**Total (common effect, 95% CI)**			**128**			**125**	**20.1%**	**--**	**−0.76 [−1.04; −0.49]**
**Total (random effect, 95% CI)**							**--**	**26.8%**	**−1.91 [−4.77; 0.96]**
Heterogeneity: $Tau^2 = 6.2670$; $Chi^2 = 65.71$, df = 2 ($P < 0.01$); $I^2 = 97\%$									
**Sedation = Clonidine**									
Nienke J. Vet et al, 2015	47.36	59.53	66	109.10	123.94	63	12.2%	9.2%	−0.64 [−0.99; −0.28]
**Sedation = Ketamine**									
Nienke J. Vet et al, 2015	10.76	19.43	66	31.40	40.20	63	12.1%	9.2%	−0.65 [−1.01; −0.30]
**Sedation = Fentanyl**									
Nienke J. Vet et al, 2015	6.29	7.72	66	3.89	5.0	63	12.6%	9.2%	0.36 [0.02; 0.71]
**Sedation = Others**									
Nienke J. Vet et al, 2015	12.15	17.70	66	18.46	28.86	63	12.7%	9.2%	−0.26 [−0.61; 0.08]
**Total (common effect, 95% CI)**			**566**			**558**	**100.0%**	**--**	**−0.50 [−0.63; −0.38]**
**Total (random effect, 95% CI)**							**--**	**100.0%**	**−1.08 [−2.03; −0.13]**

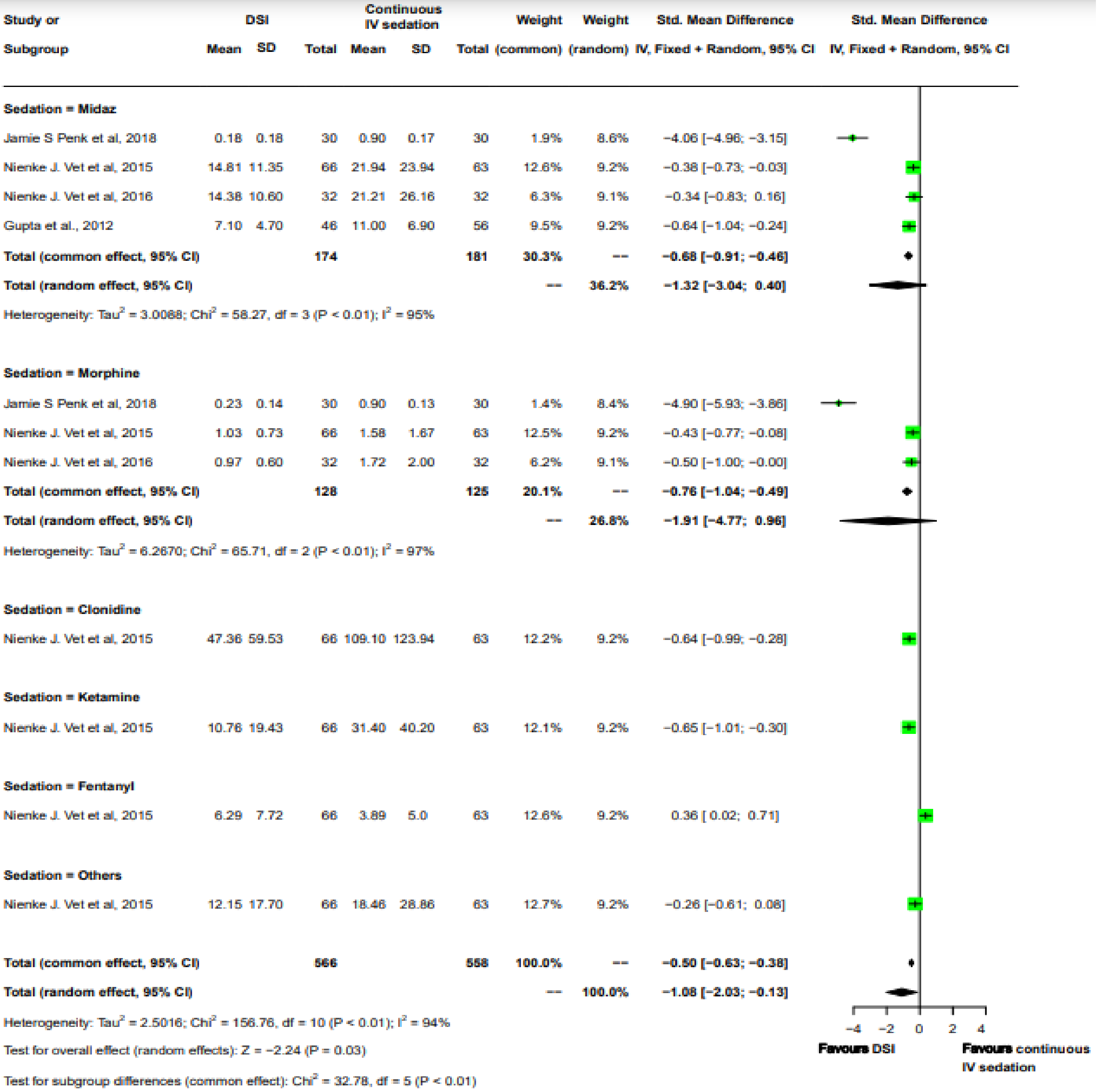

Heterogeneity: $Tau^2 = 2.5016$; $Chi^2 = 156.76$, df = 10 ($P < 0.01$); $I^2 = 94\%$

Test for overall effect (random effects): $Z = -2.24$ ($P = 0.03$)

Test for subgroup differences (common effect): $Chi^2 = 32.78$, df = 5 ($P < 0.01$)

Test for subgroup differences (random effects): $Chi^2 = 23.85$, df = 5 ($P < 0.01$)

Forest plot shows pooled effect for each sedation type and overall impact of DSI and continuous IV sedation. Each diamond represents pooled effect with 95% confidence interval of each sedation type and are based on standardized mean difference (SMD). Negative values of SMD means DSI required smaller dosages compared to continuous sedation and favours DSI.

© 2024 Shu Wen Toh T et al. *JAMA Network Open*

eFigure 2. Adverse Events in daily sedation interruption versus continuous intravenous sedation

Study	DSI Events	DSI Total	Continuous IV sedation Events	Continuous IV sedation Total	Weight (common)	Weight (random)	Risk Ratio IV, Fixed + Random, 95% CI
Nienke J. Vet et al, 2015	1	66	4	63	1.1%	2.8%	0.32 [0.05; 1.96]
Carin WM Verlaat et al, 2014	0	15	1	15	0.4%	1.0%	0.33 [0.01; 7.57]
Martha A.Q.Curley et al, 2015	41	1225	53	1224	23.8%	33.7%	0.78 [0.52; 1.15]
Martha A.Q.Curley et al, 2015	149	1225	114	1224	71.1%	53.9%	1.30 [1.04; 1.64]
Azis et al., 2016	3	18	3	22	2.1%	4.9%	1.22 [0.32; 4.68]
Gupta et al., 2012	1	46	1	56	0.8%	1.9%	1.22 [0.13; 11.30]
Gupta et al., 2012	1	46	1	56	0.8%	1.9%	1.22 [0.13; 11.30]
**Total (common effect, 95% CI)**		**2641**		**2660**	**100.0%**	**--**	**1.13 [0.93; 1.37]**
**Total (random effect, 95% CI)**					**--**	**100.0%**	**1.03 [0.76; 1.40]**

Heterogeneity: $Tau^2 = 0.0320$; $Chi^2 = 7.43$, df = 6 (P = 0.28); $I^2 = 19\%$

Test for overall effect (random effects): Z = 0.20 (P = 0.84)

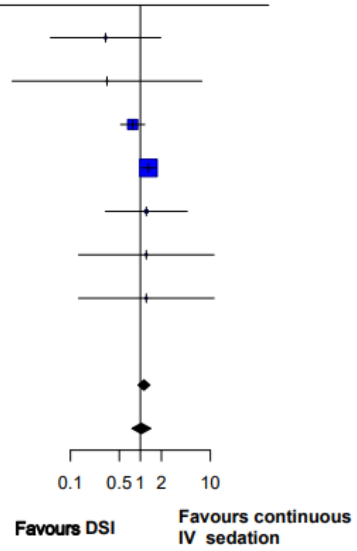

Pooled results in forest plot are expressed in terms of risk ratio (RR) with 95% confidence interval (95%CI). RR < 1 represents number of AE is smaller in DSI group compared to continuous IV sedation and hence favours DSI over continuous IV sedation.

© 2024 Shu Wen Toh T et al. *JAMA Network Open*

eFigure 3. Subgroup analysis of surgical versus medical cohort of patients receiving daily sedation interruption versus continuous intravenous sedation

Study or Subgroup	DSI Mean	DSI SD	DSI Total	Continuous IV sedation Mean	Continuous IV sedation SD	Continuous IV sedation Total	Weight (common)	Weight (random)	Mean Difference IV, Fixed + Random, 95% CI
**Surgical**									
PENK	2.67	1.15	30	4.50	4.38	30	11.7%	35.3%	−1.83 [−3.45; −0.21]
**Medical**									
CURLEY	10.51	6.98	1225	10.69	8.09	1224	86.0%	50.3%	−0.18 [−0.77; 0.42]
GUPTA	10.70	6.10	32	14.10	9.80	40	2.2%	14.4%	−3.40 [−7.10; 0.30]
**Total (common effect, 95% CI)**			**1257**			**1264**	**88.3%**	**--**	**−0.26 [−0.85; 0.33]**
**Total (random effect, 95% CI)**							**--**	**64.7%**	**−1.25 [−4.23; 1.73]**
Heterogeneity: $Tau^2 = 3.37$; $Chi^2 = 2.84$, df = 1 (P = 0.09); $I^2 = 65\%$									
**Total (common effect, 95% CI)**			**1287**			**1294**	**100.0%**	**--**	**−0.44 [−1.00; 0.11]**
**Total (random effect, 95% CI)**							**--**	**100.0%**	**−1.22 [−2.87; 0.42]**
Heterogeneity: $Tau^2 = 1.30$; $Chi^2 = 6.04$, df = 2 (P = 0.05); $I^2 = 67\%$									
Test for overall effect (random effects): Z = −1.46 (P = 0.14)									
Test for subgroup differences (common effect): $Chi^2 = 3.19$, df = 1 (P = 0.07)									
Test for subgroup differences (random effects): $Chi^2 = 0.11$, df = 1 (P = 0.74)									

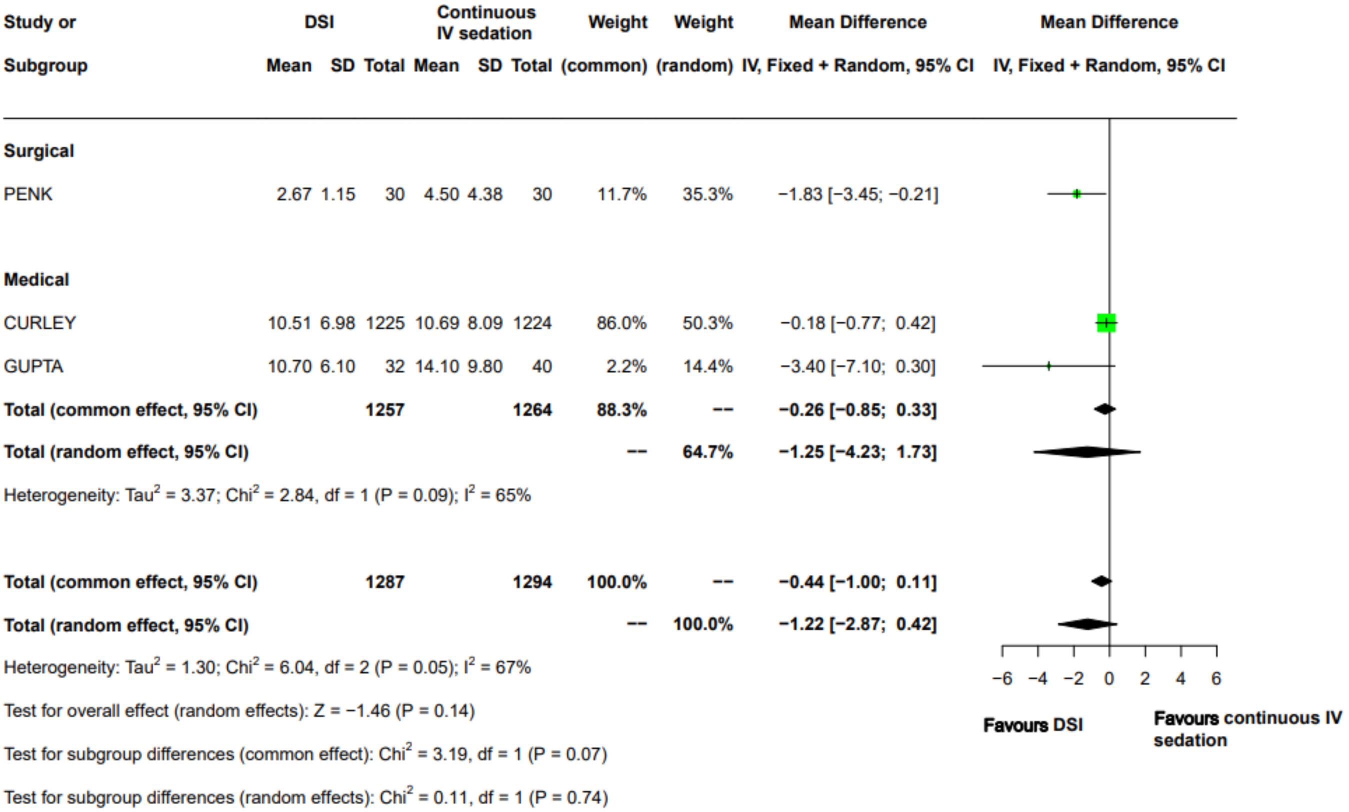

Forest plot shows pooled effect for treatment type and overall impact of DSI and continuous IV sedation. Each diamond represents pooled effect with 95% confidence interval (95% CI) of each sedation type and are based on mean difference (MD). Negative values of MD means DSI group has shorter length of ICU stay compared to continuous sedation and favours DSI.

© 2024 Shu Wen Toh T et al. *JAMA Network Open*

eFigure 4. Funnel Plot for length of stay in PICU

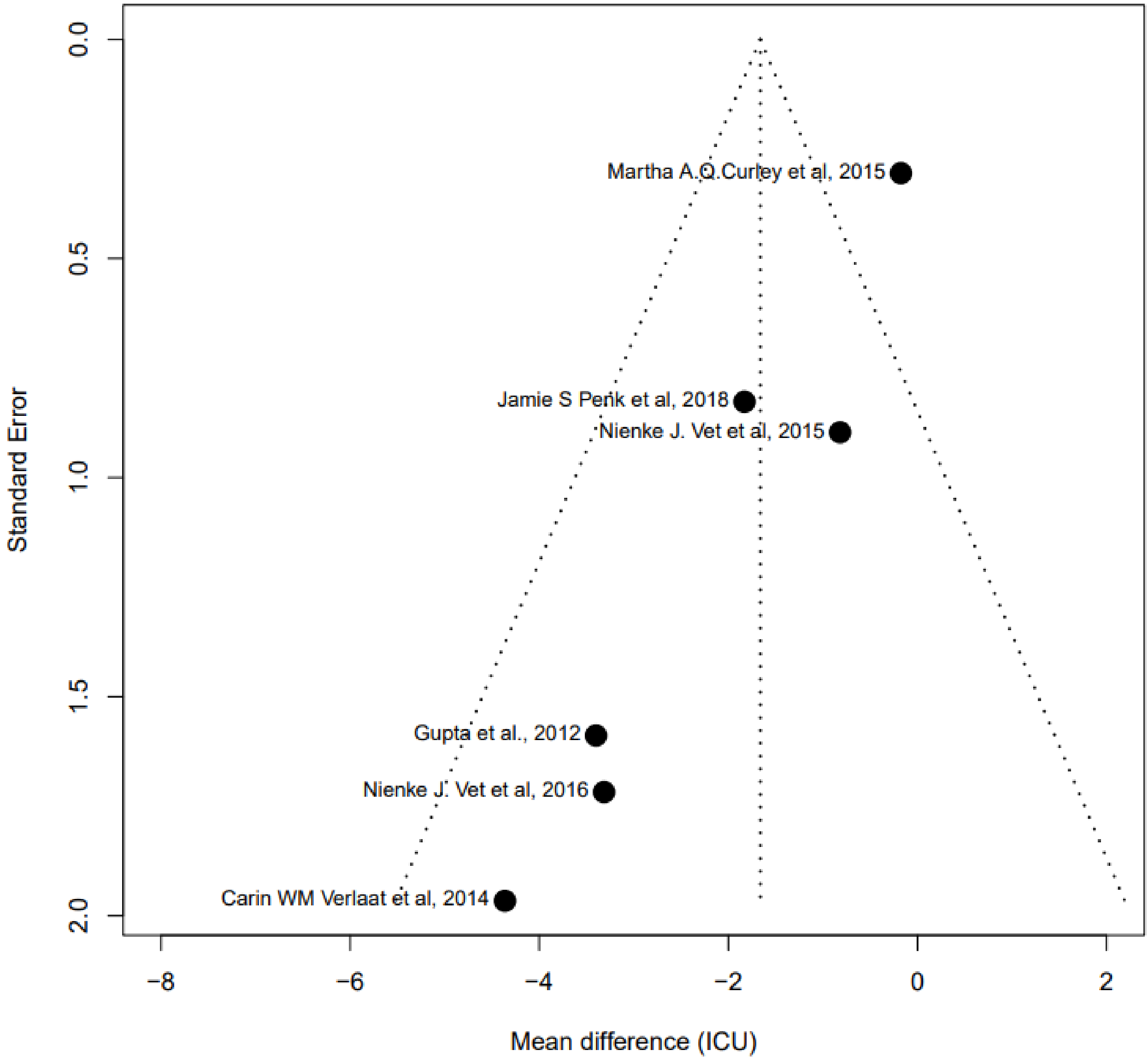

The plot displays the mean difference (x-axis) and precision (y-axis) of the mean difference. Each solid circle represents a separate study. Two slanted dotted lines in either side represent the pseudo 95% confidence intervals and the middle solid line indicates the overall effect from the meta-analysis. Small studies scatter widely at the bottom of the graph, with the spread narrowing among larger studies.

© 2024 Shu Wen Toh T et al. *JAMA Network Open*

eTable 1. Characteristics of Patients included in studies (n=2810)

Author, year	Total Sample Size	Sample Size		Male (%)		Age (months)*		Weight/kg		Primary Diagnosis		Presence of Comorbidities		Severity Score	
		Control	Intervention	Control	Intervention	Control	Intervention	Control	Intervention	Control	Intervention	Control	Intervention	Control	Intervention
Gupta et al.[1], 2012	102	56	46	71.4	67.4	49.2 (±44.4)	43.2 (±37.2)	-	-	Neuro: 40 Others (NOS[#]): 16	Neuro: 32 Others (NOS[#]): 14	-	-	PRISM 16.4±9.3	PRISM 15.3 ±5
Verlaat et al.[2], 2014	30	15	15	40	73.3	2 (0-35)	1 (0-14)	6 (5-16)	5 (3-7)	Medical: 14 (Respiratory: 9, Others[†]: 5) Surgical: 1	Medical: 13 (Respiratory: 12, Neuro: 1) Surgical: 2	-	-	PRISM 13 (10-18) PIM[c] 7 (3-19)	PRISM 16 (9-23) PIM 7 (5-16)
Curley et al.[3], 2015	2449	1224	1225	55.6	54.4	31.2(7.2-110.4)	16.8(3.6-84)	-	-	Respiratory: 1224	Respiratory: 1225	Neuro: 208 Non-neuro: 542	Neuro: 217 Non-neuro: 488	PRISM[b] 8 (5-13.5)	PRISM 6 (3-11)
Vet et al.[4], 2016	129	63	66	65.1	57.6	2.7 (1.3-14)	2.8 (1.1-17.1)	4.6 (3.7-11)	5 (3.7-10)	Medical: 50 (Respiratory: 40, Other medical diagnoses[†]: 10) Surgical: 9 Others (NOS[#]): 4	Medical: 57 (Respiratory: 47, Other medical diagnoses[†]: 10) Surgical: 8 Others (NOS[#]): 1	-	-	PELOD 11 (11-20) PRISM 16 (11-21) PIM 3.2 (1.5-7.6)	PELOD 11 (8-20) PRISM 16.5 (13-24) PIM 4.3 (1.6-10)
Azis et al.[5], 2016	40	18	22	72.2	72.7	54 (2.4-183.6)	12 (0.96-168)	-	-	Medical: 12 (Respiratory: 2 Neuro: 10) Others (NOS[#]): 6	Medical: 17 (Respiratory: 6 Neuro: 11) Others (NOS[#]): 5	-	-	PELOD[a] 11 (2-31)	PELOD 11 (2-31)
Penk et al.[6], 2018	60	30	30	43.3	40	14.8 (±12.1)	16 (±12.8)	-	-	Surgical: 30	Surgical: 30	Non-neuro: 5 None: 25	Non-neuro: 4 None: 26	-	-

[a]PELOD: Pediatric Logistic Organ Dysfunction, [b]PRISM: Pediatric Risk of Mortality, [c]PIM: Pediatric Index of Mortality

*Variable may be represented in mean (± standard deviation) or median (interquartile range)

[†]Other medical diagnoses include cardiac disorders, sepsis or septic shock and oncological diagnoses

[#]NOS: not otherwise specified

© 2024 Shu Wen Toh T et al. *JAMA Network Open*

eTable 2. Daily sedation interruption (DSI) versus continuous intravenous (IV) sedation by dosing regimen or by distribution across different sedative agents (n=2810)

Author, year	Sedation Protocol		Sedation Driven by		Midazolam		Morphine		Dexmedetomidine		Clonidine		Ketamine		Fentanyl		Others	
	Control	Intervention	Control	Intervention	Control	Intervention	Control	Intervention	Control	Intervention	Control	Intervention	Control	Intervention	Control	Intervention	Control	Intervention
Gupta et al.[1], 2012	56	46	-	-	0.1-0.3mg/kg/hr	initial bolus 0.1-0.3mg/kg over 2-3mins, followed by infusion at 0.1mg/kg/h, titrated to attain a Ramsay score of 3-4 with daily interruption at 8am	0.01-0.03mg/kg/hr	0.01–0.03 mg/kg/hr with daily interruption at 8am	-	-	-	-	-	-	-	-	-	-
Verlaat et al.[2], 2014	15	15	-	-	15 (0.2 [0.1-0.28] mg/kg/hr)	15 (baseline use 0.12 [0.1-0.2] mg/kg/hr)	15 (10 [8-20] mcg/kg/hr)	15 (baseline 9mcg/kg/hr [7-11])	-	-	-	-	-	-	-	-	-	-
Curley et al.[3], 2015*	1224	1225	Nurse	Nurse	1009	1087	210	782	596	287	163	149	368	261	989	431	Lorazepam: 20	Lorazepam: 128
Vet et al.[4], 2016*	63	66	Nurse	Nurse	63	66	52	54	-	-	11	13	17	9	28	34	Propofol: 29	Propofol: 24
Azis et al.[5], 2016	18	22	-	-	initial 0.2-0.3mg/kg bolus, then infusion at 0.1mg/kg/hr (increased to 0.3mg/kg/hr) to achieve COMFORT pain scale score of 11-22	-	-	-	-	-	-	-	-	-	-	-		
Penk et al.[6], 2018	30	30	Nurse and doctor	Nurse and doctor	30 (0.03mg/kg/hr)	-	30 (0.03mg/kg/hr)	-	-	-	-	-	-	-	-	-	-	-

*Dosing regimen for sedatives and interruption protocols described in further detail in respective papers

© 2024 Shu Wen Toh T et al. *JAMA Network Open*

eTable 3. Patient Outcomes in both daily sedation interruption (DSI) and continuous intravenous (IV) sedation groups (n=2810)

Author, year	Duration of MV (days)*		PICU LOS (days)*		Hospital LOS (days)*		Sedation Score*		Total Sedation Dose*		Withdrawal Score*		Adverse Events[b]		Mortality	
	Control	Intervention	Control	Intervention	Control	Intervention	Control	Intervention	Control	Intervention	Control	Intervention	Control	Intervention	Control	Intervention
Gupta et al.[1], 2012	10.3 (±8.4)	7.1 (±4.8)	14.1 (±9.8)	10.7 (±6.1)	-	-	-	-	Midazolam: 11 (±6.9) mg/kg/day	Midazolam: 7.1 (±4.7) mg/kg/day	-	-	• Accidental extubation: 1<br>• Pneumothorax: 7	• Accidental extubation: 1<br>• Pneumothorax: 5	15/56 (26.8%)	12/46 (26.1%)
Verlaat et al.[2], 2014	9 (5-14)	4 (3-8)	10 (7-15)	6 (4-9)	-	-	-	-	-	-	-	-	• Accidental extubation: 1	• Removed IV access: 1	0/15 (0%)	0/15 (0%)
Curley et al.[3], 2015	6.5 (3.7-12.1)	6.5 (4.1-11.2)	9.6 (5.7-16.6)	9.6 (6.2-15.6)	-	-	-	-	-	-	-	-	• Accidental extubation: 0.53 (n.o of events/100 ventilator days)<br>• Withdrawal syndrome: 114<br>• Inadequate pain control: 174<br>• Extubation failure: 104<br>• Post-extubation stridor: 55<br>• Catheter-associated bloodstream infections: 0.77 (n.o of events/1000 central line days)<br>• Ventilator-associated pneumonia: 0.77 (n.o events/1000 ventilator days)<br>• Immobility related ulcers: 19<br>• New tracheostomy: 33	• Accidental extubation: 0.41 (n.o of events/100 ventilator days)<br>• Withdrawal syndrome: 149<br>•Inadequate pain control: 195<br>• Extubation failure: 97<br>• Post-extubation stridor: 88<br>• Catheter-associated bloodstream infections: 0.86 (n.o of events/1000 central line days)<br>• Ventilator-associated pneumonia: 0.53 (n.o events/1000 ventilator days)<br>• Immobility related ulcers: 5<br>• New tracheostomy: 16	63/1224 (5.2%)	47/1225 (3.8%)

Author, year	Duration of MV (days)*		PICU LOS (days)*		Hospital LOS (days)*		Sedation Score*		Total Sedation Dose*		Withdrawal Score*		Adverse Events[b]		Mortality	
	Control	Intervention	Control	Intervention	Control	Intervention	Control	Intervention	Control	Intervention	Control	Intervention	Control	Intervention	Control	Intervention
Vet et al.[4], 2016	5.2 (3.6-9)	5.1 (3.7-7.1)	7.4 (5.3-12.8)	6.9 (5.2-11)	15.7 (9.3-33.2)	13.3 (8.6-26.7)	COMFORT B: 12 (10-14)	COMFORT B: 12 (11-15)	•Midazolam: 17 (8.2-39.8) mg/kg<br>• Morphine: 1.16 (0.65-2.86) mg/kg<br>• Clonidine: 75.7 (41.2-204.8) mcg/kg<br>• Ketamine: 35.63 (3.11-56.17) mg/kg<br>• Fentanyl: 2.3 (1.2-7.9) mcg/kg<br>• Propofol: 10.8 (2.6-40.7) mg/kg	• Midazolam: 14.1 (7.6-22.6) mg/kg<br>• Morphine: 0.92 (0.6-1.56) mg/kg<br>• Clonidine: 47.4 (8-86.7) mcg/kg<br>• Ketamine: 4.51 (0.52-26.2) mg/kg<br>• Fentanyl: 4.1 (2.1-12.3) mcg/kg<br>• Propofol: 6.5 (2.8-26.2) mg/kg	SOS[d] 1 (1-2.8)	SOS 1 (0.5-2)	• Accidental extubation: 4<br>• Oversedation: 1	• Accidental extubation: 1<br>• Need for restraints: 1	0/63 (0%)	6/66 (9.1%)
Azis et al.[5], 2016	2.5 (1.3-5.5)	1.7 (1.3-4)	-	3.5	-	-	-	-	-	-	-	-	• Accidental extubation: 3<br>• Hypotension: 1	• Accidental extubation: 3<br>• Hypotension:2	-	-
Penk et al.[6], 2018	-	-	4.50 (±4.38)	2.67 (±1.15)	8.37 (±7.68)	4.9 (±2.52)	FLACC mean 1.34 (IQR 0.62-2.02), median 0.00 (IQR 0-2)	FLACC mean 1.58 (IQR 0.8-2.35), median 1.00 (IQR 0-2.62)	• Midazolam: 0.9 (±0.17) mg/kg<br>• Morphine: 0.9 (±0.13) mg/kg	• Midazolam: 0.18 (±0.18) mg/kg<br>• Morphine: 0.23 (±0.14) mg/kg	-	-	-	-	-	-

[d] Sophia Observation Withdrawal Symptoms Scale

[b] absolute numbers of adverse events unless stated otherwise

*Variable may be represented in mean (± standard deviation) or median (interquartile range)

© 2024 Shu Wen Toh T et al. *JAMA Network Open*

eReferences:

1. Gupta K, Gupta VK, Jayashree M, Singhi S. Randomized controlled trial of interrupted versus continuous sedative infusions in ventilated children. *Pediatr Crit Care Med*. Mar 2012;13(2):131-5. doi:10.1097/PCC.0b013e31820aba48
2. Verlaat CW, Heesen GP, Vet NJ, et al. Randomized controlled trial of daily interruption of sedatives in critically ill children. *Paediatr Anaesth*. Feb 2014;24(2):151-6. doi:10.1111/pan.12245
3. Curley MA, Wypij D, Watson RS, et al. Protocolized sedation vs usual care in pediatric patients mechanically ventilated for acute respiratory failure: a randomized clinical trial. *JAMA*. Jan 27 2015;313(4):379-89. doi:10.1001/jama.2014.18399
4. Vet NJ, de Wildt SN, Verlaat CW, et al. A randomized controlled trial of daily sedation interruption in critically ill children. *Intensive Care Med*. Feb 2016;42(2):233-44. doi:10.1007/s00134-015-4136-z
5. Azis H, Triratna S, Bahar E. Continuous sedation vs. daily sedation interruption in mechanically-ventilated children. *Paediatrica Indonesiana*. 2016;56(1):19-23.
6. Penk JS, Lefaiver CA, Brady CM, Steffensen CM, Wittmayer K. Intermittent Versus Continuous and Intermittent Medications for Pain and Sedation After Pediatric Cardiothoracic Surgery; A Randomized Controlled Trial. *Crit Care Med*. Jan 2018;46(1):123-129. doi:10.1097/CCM.0000000000002771

© 2024 Shu Wen Toh T et al. *JAMA Network Open*
