# Supplementary material for: Daily Sedation Interruption vs Continuous Sedation in Pediatric Patients Receiving Mechanical Ventilation: A Systematic Review and Meta-analysis
Source: JAMA Netw Open. 2024 Aug 7;7(8):e2426225. doi: 10.1001/jamanetworkopen.2024.26225 (PMC11307139; doi:10.1001/jamanetworkopen.2024.26225)
Supplement: Supplement 2. — Data Sharing Statement [file jamanetwopen-e2426225-s002.pdf]

Data Sharing Statement

Shu Wen Toh. Daily Sedation Interruption vs Continuous Sedation in Pediatric Patients Receiving Mechanical Ventilation. *JAMA Netw Open*. Published August 07, 2024. doi:10.1001/jamanetworkopen.2024.26225

Data
Data available: Yes
Data types: Deidentified participant data
How to access data: NIL
When available: With publication

Supporting Documents
Document types: None

Additional Information
Who can access the data: Anyone requesting the data
Types of analyses: For purposes related to research intent
Mechanisms of data availability: After approval of a proposal and with signed data access agreement
